# Assessing the Educational Impact of Dissection Competitions on Medical Students

**DOI:** 10.7759/cureus.94071

**Published:** 2025-10-07

**Authors:** Ujwala Bhanarkar, Grace Suganya, Biswabina Ray

**Affiliations:** 1 Department of Anatomy, All India Institute of Medical Sciences, Kalyani, Kalyani, IND

**Keywords:** anatomy education, dissection competition, dissection impact, medical education, problem-based learning

## Abstract

Background

Dissection competitions have recently emerged as a novel approach integrating traditional dissection practices with a competitive, problem-based learning model. This approach not only reinforces anatomical knowledge through hands-on experience but also introduces elements of competition to create a more stimulating and engaging learning environment. Hence we aimed at evaluating the educational impact of dissection competitions among first-year MBBS students.

Methods

The study was conducted among 158 first year MBBS students at AIIMS Kalyani using a Google Form questionnaire. A mixed-methods research design was employed in this study. Quantitative data were collected through structured feedback forms that measured various aspects of the competition, including overall satisfaction, perceived learning outcomes, and the relevance of the competition format to students' studies. In addition, qualitative data were gathered from open-ended responses, providing deeper insights into the students' experiences, perceived benefits, and suggestions for future improvements.

Results

The findings from the structured feedback and thematic analysis revealed that dissection competitions significantly enhance students' understanding of anatomical concepts. Additionally, the competitive and collaborative nature of the event helped foster critical skills such as teamwork, effective communication, and problem-solving, which are crucial for future medical professionals.

Conclusion

The findings of this study would provide valuable insights into the benefits of integrating dissection competitions into the medical curriculum. The results suggest that such competitions are not only effective in enhancing anatomy education but also in developing essential skills needed for medical practice.

## Introduction

Anatomy education has traditionally relied on passive learning methods such as lectures, textbooks, and supervised cadaver dissections [1]. While these methods provide a fundamental understanding of human anatomy, they often lack the interactivity required to fully engage students and stimulate deeper learning [2]. Dissection holds a pivotal role in the Bachelor of Medicine and Bachelor of Surgery (MBBS) curriculum as it serves as a cornerstone for understanding human anatomy, bridging the gap between theoretical knowledge and practical application [3,4]. It provides medical students with a unique, hands-on experience that allows them to explore the three-dimensional complexity of the human body, offering insights that cannot be fully captured through textbooks, lectures, or digital models alone. By dissecting cadavers, students gain a deep appreciation of anatomical structures, their spatial relationships, and variations, which is crucial for clinical practice. Dissection enhances observational and tactile skills, fosters critical thinking, and encourages an inquiry-based approach to learning [5]. It also cultivates professionalism, teamwork, and ethical considerations, as students reflect on the significance of working with human bodies. Moreover, dissection provides a foundation for clinical skills, such as palpation, surgery, and understanding pathologies, making it an indispensable component of medical education that prepares students for real-life scenarios in patient care. Thus, integrating dissection competition into the MBBS curriculum ensures a comprehensive and holistic education, laying a strong foundation for future medical practitioners.

In recent years, there has been a growing emphasis on incorporating innovative teaching strategies that promote active learning, such as problem-based learning (PBL), team-based learning (TBL), and simulation-based education [6,7]. These methodologies encourage students to engage more deeply with the material, thereby enhancing their understanding and retention of complex subjects [8].

Dissection competitions represent a novel approach to anatomy education, combining the traditional practice of cadaver dissection with elements of competition to foster a more interactive and stimulating learning environment [9-11]. At AIIMS Kalyani, a dissection competition was organized to test the feasibility and educational value of such an approach. The competition aimed to enhance students' anatomical knowledge through hands-on learning, foster teamwork and collaboration, and encourage critical thinking and problem-solving. Following the competition, structured feedback was collected from participants to evaluate its effectiveness as a pedagogical tool. 

The primary objective of this study was to evaluate the educational value of dissection competitions on medical students understanding of anatomy and overall learning experience. Specifically, the study seeks to assess students' perceptions of the competition, including its perceived value, challenges, and learning outcomes. Additionally, the study also aimed to identify key strengths and potential areas for improvement based on participant feedback and explore the role of competitive, active learning in enhancing traditional anatomical education.

## Materials and methods

The study was carried out after getting ethical clearance from the Institutional Ethical Committee of AIIMS Kalyani (Ref. no. IEC/AIIMS/Kalyani/certificate/2024/424).

Details of the dissection competition

The dissection competition at AIIMS Kalyani was first introduced for the 2022 MBBS batch, aimed at fostering an interactive, hands-on learning experience in anatomy education. 

For the 2022 MBBS batch, students interested in participating were encouraged to enrol voluntarily. Out of a total of 125 students, 33 (26%) students showed interest and took part in the event. These 33 students were strategically divided into 15 smaller groups, each team consisting of two to three participants to ensure effective collaboration and engagement. The competition was structured to span over three days, with each session lasting two hours, held during the regular dissection classes. The dissection topic was allotted to the groups by picking a lot a day before the competition. Thus the students knew the topic before coming for the competition and were given time of one day to prepare for the dissection.

To maintain a fair and objective evaluation process, a structured format was meticulously developed for judging the participants' performance. The evaluation criteria included various aspects such as anatomical knowledge, precision of dissection, clarity of presentation, and teamwork. A panel of 10 esteemed faculty members from the Department of Anatomy served as judges, bringing in their expertise to assess the participants. At the end of the competition, the top three teams were declared as winners and were awarded prizes in recognition of their outstanding performance.

Following a positive response and educational benefits observed from the first competition, a similar event was organized in the following year for the 2023 MBBS batch. Unlike the previous year, the competition saw full participation from the entire batch of 125 students, demonstrating increased interest and enthusiasm for this innovative learning approach. To manage the larger number of participants, the students were divided into 25 groups, each comprising five students.

The format for the 2023 competition was further refined to enhance learning outcomes and provide a more comprehensive educational experience. Each group was assigned one of 25 diverse topics in anatomy, ensuring a broad coverage of subjects and promoting in-depth exploration of specific areas (Table 1). The topics were allotted based on the availability of the cadaver/specimens. To maintain uniformity of the rubric, the topics were allotted by picking the lot a day before the competition and the judging criteria was informed to the students beforehand and one day's time was given to the students for preparation. All the topics were taught already to the undergraduate students and the competition was organised at the end of their curriculum. These topics are routinely dissected in our department. A mentor was allotted for three groups each to guide the students for dissection for the presentation of specimens and to clear their doubts. The competition was again conducted over three days, with each session lasting for two hours, integrated into the regular dissection classes to maximize student learning opportunities.

**Table 1:** Distribution of groups and dissection topics alloted to the 2023 batch

Groups	Topics allotted
Group 1	Pharynx+esophagus and larynx+trachea
Group 2	Lumbo-sacral spine (dorsal aspect) along with associated ligaments
Group 3	Lumbo-sacral spine (ventral and lateral aspect) along with roots of origin of plexus
Group 4	Infratemporal fossa (branches of maxillary artery - right side)
Group 5	Prevertebral region of cervical spine and course of vertebral artery in neck (both sides)
Group 6	Coeliac trunk and its branches
Group 7	Anterior compartment of leg with dorsum of foot (right side): muscles, tendons, and neurovascular structures
Group 8	All joints of foot along with associated ligaments
Group 9	Branches of facial nerve in face (left side)
Group 10	Extrahepatic biliary apparatus along with pancreatic duct system and drainage apparatus
Group 11	Nerves in the palm (left palm)
Group 12	Interosseus membrane with nerve and vessels (right forearm)
Group 13	Inguinal region (left side)
Group 14	Pelvic floor muscles: male pelvis (superior and inferior view)
Group 15	Small and large intestine: Comparison (luminal differences, wall features)
Group 16	Prevertebral region of cervical spine and proximal part of cervical and brachial plexus
Group 17	Deep palmar arch and branches (right palm)
Group 18	Intestinal arterial arcade
Group 19	Coronary arteries: Origin, course and branches
Group 20	Posterior abdominal wall (Retroperitoneal structures)
Group 21	All joints of hand along with associated ligaments (left side)
Group 22	Intercostal nerve: From spinal segment till as distal as possible in the given specimen (on both sides)
Group 23	Intercostal space with contents, their relation and sympathetic chain (on both sides)
Group 24	Lateral and posterior compartment of leg: muscles, tendons, neurovascular structures, and interosseus membrane
Group 25	Complete arterial supply of thyroid gland (neck)

Evaluation criteria and judges 

Similar to the previous year, a structured judging format was employed to evaluate the performance of the participants, ensuring consistency and fairness in assessment. The same panel of 10 faculty members from the Department of Anatomy served as judges, bringing their expertise to the evaluation process. The judging criteria remained focused on key aspects such as anatomical knowledge, accuracy in dissection, presentation skills, and teamwork. 

A structured evaluation sheet was developed to ensure objective and consistent assessment across all groups. The evaluation criteria included five main components. Completeness (four marks): Whether the given topic was completely dissected or only partially dissected. Relevance (four marks): Whether the dissected part or structure was relevant to the assigned topic. Clarity (four marks): Whether the structures were neatly dissected with minimal damage to the surrounding or adjacent regions. Identification/labelling (four marks): Whether the dissected structures were properly labelled and correctly identified by the group. Display (four marks): How the group presented their dissected specimen in front of the evaluators.

**Figure 1:**
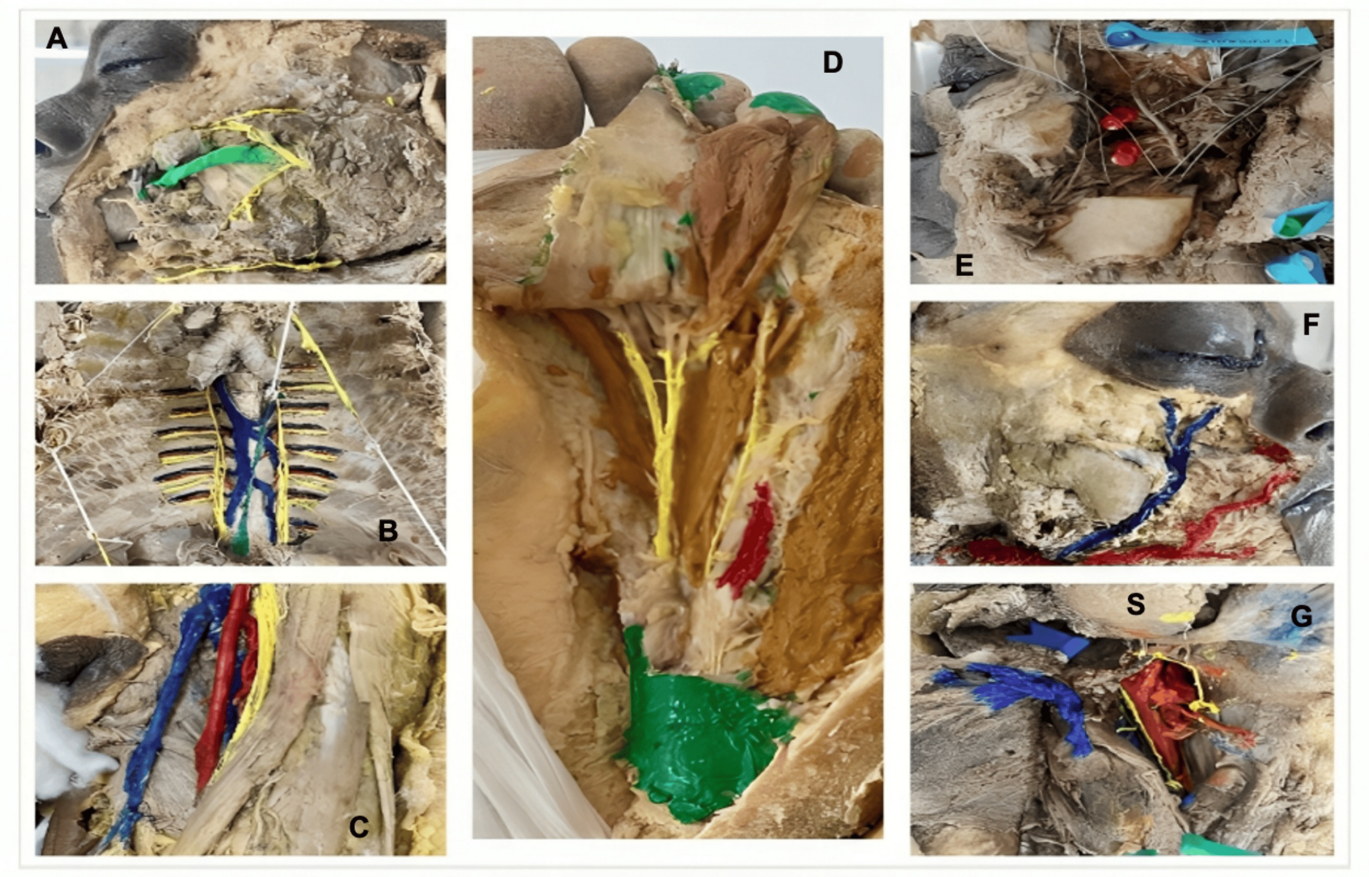
Examples of dissected specimens by different groups in competition A: Dissection of branches of facial nerve in face. Yellow - branches of facial nerve in face; green - parotid duct B: Dissection of the azygos system. Red – posterior intercostal arteries; blue - posterior intercostal veins and azygos system; yellow - posterior intercostal nerves C: Dissection of contents of femoral triangle. Red - femoral artery; blue - femoral veins; yellow - femoral nerve. D: Dissection of second layer of sole. Brown - muscles in second layer of sole; red - lateral plantar artery; yellow - medial and lateral plantar nerves; green – part of plantar aponeurosis E: Dissection of infratemporal fossa. Two red pins - two heads of lateral pterygoid F: Dissection of facial artery and vein in face. Red - facial artery; blue - facial vein G: Dissection of contents of carotid triangle. Red - carotid vessels; S - submandibular gland

Each group was scored on a total of 20 marks based on these criteria. The judging panel ensured that the evaluations were carried out objectively to maintain fairness and provide constructive feedback to all the participants. The top three teams were again recognized for their exceptional performance and were awarded prizes. The dissected specimens from different groups, along with their presentation, are given in Figure 1.

Study design 

This study employed a mixed-methods approach, combining both quantitative and qualitative data to provide a comprehensive evaluation of the educational impact of the dissection competition. A total of 158 students from the 2022 and 2023 MBBS batches from AIIMS Kalyani who participated in the dissection competition were included in the study. The feedback collection process was voluntary, and all participants provided informed consent before participation.

Validation of the questionnaire

The questionnaire was first given to the postgraduates (seven students) in the department for confirming its validity. Then, it was distributed to the undergraduates after making the necessary changes.

Data collection

Data was collected using a structured online feedback Google Form, distributed immediately after the competition. The feedback form was designed to capture both quantitative data through closed-ended questions and qualitative insights through open-ended questions. Closed-ended questions focused on various aspects of the competition, including overall satisfaction, perceived learning outcomes, relevance to their studies, and the effectiveness of the competition format. Open-ended questions allowed students to provide detailed feedback on their experiences, highlighting what they found most valuable, the challenges they faced, and their suggestions for improvement.

Data analysis

Quantitative data from closed-ended questions were represented as a percentage. Qualitative data from open-ended responses were analyzed using thematic analysis, where responses were coded and categorized into recurring themes that provided deeper insights into the students' experiences. This mixed-methods approach allowed for a comprehensive understanding of both the measurable outcomes and the nuanced experiences of the participants.

## Results

The responses to the closed-ended questions were analyzed using descriptive statistics. These questions were structured on a Likert scale, where students rated their agreement or satisfaction with various statements related to the competition (Table 2).

**Table 2:** Structured close ended questions and their responses from students using the Likert scale

Questions	Strongly Agree	Agree	Neutral	Disagree	Strongly Disagree
	N=158 (%)				
Did you find the topic allotted for dissection appropriate	63 (40)	55 (35)	24 (15)	8 (5)	8 (5)
Time given for dissection was adequate	47 (30)	71 (45)	16 (10)	16 (10)	8 (5)
Cadaver given for dissection was good	79 (50)	47 (30)	16 (10)	8 (5)	8 (5)
Positioning of the cadaver eased the process of dissection	71 (45)	63 (40)	16 (10)	5 (3)	3 (2)
Structures were easily accessible	55 (35)	71 (45)	24 (15)	8 (5)	0 (0)
It was easy to use the dissection instruments	47 (30)	79 (50)	16 (10)	11 (7)	5 (3)
Felt confident before starting the dissection	39 (25)	63 (40)	32 (20)	16 (10)	8 (5)
Identification of the structures during dissection was easy	32 (20)	72 (50)	24 (15)	16 (10)	8 (5)
Using acrylic paint to highlight the structures would help future learning	90 (55)	4 (30)	16 (10)	5 (3)	3 (2)
Prior reading (theoretical knowledge) helped during dissection	94 (60)	40 (25)	16 (10)	5 (3)	3 (2)
This dissection competition increased my interest in anatomy	102 (65)	32 (20)	16 (10)	5 (3)	3 (2)
The competition will be helpful for future anatomy examinations	78 (50)	48 (30)	24 (15)	5 (3)	3 (2)
Dissection deepened the understanding of anatomy of a particular area	110 (70)	32 (20)	8 (5)	5 (3)	3 (2)
The allotted time for dissection was inadequate	16 (10)	24 (15)	32 (20)	54 (35)	32 (20)
The smell and effect of formalin reduced the speed of my dissection	24 (15)	40 (25)	46 (30)	32 (20)	16 (10)
Conducting similar events in the future will be helpful	126 (80)	24 (15)	5 (3)	3 (2)	0 (0)
I shall encourage my juniors to participate in similar events	118 (75)	32 (20)	5 (3)	3 (2)	0 (0)

Quantitative results

Based on the feedback received from the participants of the dissection competition, several key insights were gathered through a series of structured questions. The quantitative analysis demonstrated high overall satisfaction among participants, with a mean satisfaction score of 4.5 out of 5. Most students reported that the competition significantly enhanced their understanding of anatomical structures, particularly in areas they dissected. About 85% of the participants indicated an improved grasp of complex anatomical relationships and spatial arrangements as a result of the hands-on, competitive nature of the event. Additionally, 90% of the students felt that the competition was highly relevant to their anatomy studies, emphasizing that the interactive, hands-on experience allowed them to apply theoretical knowledge in a practical setting. The competitive format of the event was seen as particularly effective, with 80% of respondents agreeing that it increased their engagement and motivation to learn. Overall, the quantitative results suggest that the dissection competition was well-received, deemed beneficial for learning, and appreciated for its innovative approach to enhancing anatomy education among MBBS students.

Qualitative results

Thematic analysis of the qualitative feedback identified several key themes. One prominent theme was Enhanced Engagement, where students expressed that the competitive element of the competition made the learning process more dynamic and interesting, thereby increasing their motivation to study and perform well. Another theme was Teamwork and Collaboration, with many participants highlighting the benefits of working in teams, which not only helped in dividing the workload but also in learning from peers, fostering a collaborative learning environment. The theme of Critical Thinking and Problem-Solving emerged as well, as the competition format encouraged students to apply their anatomical knowledge in creative and practical ways, such as creating innovative presentations or answering scenario-based questions posed by the judges. However, participants also provided constructive feedback, suggesting improvements like more guidance from mentors during the dissection process and increased preparation time for more complex dissections.

## Discussion

The present study explores the educational benefits of integrating dissection competitions into the anatomy curriculum for MBBS students. The results demonstrate that such competitions are not only effective in enhancing anatomical understanding but also in fostering enthusiasm, critical thinking, and collaboration among students. The positive reception among the participants, as evidenced by the high percentage of students who found the competition beneficial, aligns with a growing body of literature that emphasizes the importance of active learning in medical education.

Several studies have highlighted the significance of traditional cadaveric dissection in medical education. A study by Lempp (2005) [11] and Winkelmann et al. (2007) [12] emphasized that cadaveric dissection allows students to gain a comprehensive understanding of the spatial relationships and complexities of human anatomy, which is critical for clinical practice. Our findings support this perspective, with 75% of the students agreeing that the topics allotted for dissection were appropriate and 85% indicating that the competition increased their interest in learning anatomy. This suggests that dissection competitions can serve as a valuable complement to traditional dissection classes, providing a more engaging and dynamic learning experience that reinforces anatomical concepts.

Comparatively, modern teaching methods such as virtual dissection tools and 3D models have gained popularity in recent years. Studies such as those by Peterson and Mlynarczyk (2016) [13], Choi-Lundberg DL et al. (2016) [14] and Hisley (2023) [15] suggest that while these methods offer interactivity and accessibility, they lack the tactile feedback and three-dimensional realism of actual cadaveric dissection. In our study, students overwhelmingly favoured the hands-on experience provided by dissection competitions. A significant 75% of participants strongly agreed that they would encourage their juniors to participate in similar events, which reflects the unique value they perceive in direct interaction with human tissue, something that virtual methods cannot fully replicate. This reinforces the argument that despite technological advancements, traditional cadaveric dissection remains a cornerstone of anatomy education.

Moreover, the competitive aspect of the event seems to enhance motivation and active participation among students. Similar findings have been reported in studies by Wilkinson et al. (2019) [16], Verma et al. (2024) [17] and Rudolphi-Solero et al. (2021) [18] who observed that the integration of competitive elements, such as simulation-based competitions and anatomy quizzes, positively impacts student engagement and learning outcomes. Our study supports this view, with 65% of the students strongly agreeing that the competition increased their interest in anatomy. This suggests that the competitive format, combined with the tactile experience of dissection, may stimulate intrinsic motivation and foster a deeper connection with the subject matter.

However, while the majority of feedback was positive, there were some concerns raised by the participants regarding the organization of the competition. About 5% of students disagreed that the time allotted for dissection was adequate, suggesting a need for more flexible or extended time slots to allow a thorough exploration and understanding of anatomical structures. Additionally, 3% of students reported that the formalin used for preservation caused irritation or discomfort, which hindered their dissection performance. This is consistent with the findings of Tiruneh (2021) [19] and Getachew (2014) [20] who reported that formalin exposure can cause significant discomfort and affect the dissection experience. Addressing these concerns could involve providing more ventilation, offering protective gear, or exploring alternatives to formalin to enhance the comfort and efficiency of future dissection sessions.

The use of dissection competitions as a teaching tool in medical education also provides an opportunity to cultivate soft skills such as teamwork, communication, and problem-solving. These skills are vital for future clinical practice, as highlighted by Mohammed et al. (2024) [21], who noted that problem-based learning (PBL) environments in medical education contribute to the holistic development of medical students. In this context, dissection competitions offer a unique blend of PBL and traditional learning, encouraging students to work collaboratively while navigating the complexities of human anatomy. This dual approach not only reinforces anatomical knowledge but also prepares students for real-world clinical challenges where teamwork and critical thinking are paramount.

Furthermore, the overwhelmingly positive response to the idea of conducting similar events in the future endorsed by 95% of the participants suggests that dissection competitions can be effectively integrated into the anatomy curriculum as a regular feature. Future studies could explore longitudinal data to assess how participation in such competitions impacts long-term knowledge retention, exam performance, and clinical skills development. Moreover, the research could also investigate how variations in competition format, such as time constraints or topics covered, might influence student learning outcomes and engagement.

Limitations of the study

Sample Size and Generalizability

The study was conducted with a limited sample size of 178 students from the 2022 and 2023 MBBS batches at AIIMS Kalyani. Although the sample provided meaningful data for this specific context, the findings may not be generalizable to all medical students or institutions. Future studies involving multiple institutions with a larger and more diverse sample size could provide more generalizable results.

Self-Reported Data

The data for this study were collected through self-reported feedback from students, which is inherently subject to biases such as social desirability bias and recall bias. Students may have overestimated their positive experiences or provided responses they believed were expected by their instructors.

Lack of Longitudinal Follow-Up

The study captures the immediate impact of the dissection competition on students' engagement and understanding of anatomy. A longitudinal study design, involving follow-up assessments several months after the competition, would help determine whether the benefits observed are sustained over time and translate into better performance in future anatomy courses or clinical practice.

Limited Assessment of Skill Development

Although the competition aimed to foster critical thinking, teamwork, and problem-solving, the study did not include an objective assessment of these skills. Evaluating the development of these competencies through standardized tools or rubrics would provide a more comprehensive understanding of the competition's impact on students' professional growth.

Subjective Nature of Feedback Analysis

The qualitative feedback analysis relied on thematic analysis, which, although systematic, is inherently subjective. The interpretation of themes could be influenced by researchers' biases. Employing multiple analysts and cross-validation techniques could enhance the reliability of qualitative findings.

Limited Scope of Anatomical Topics Covered

Although the study included a diverse range of anatomical topics for the competition, it is possible that not all key areas of anatomy were covered comprehensively. Future studies could aim for a more exhaustive inclusion of topics, potentially rotating them annually to ensure a broader scope of learning.

## Conclusions

This study adds to the growing evidence supporting the integration of innovative, student-centered learning methodologies in medical education. Dissection competitions represent a promising avenue for enhancing anatomical education by combining the strengths of traditional dissection with the benefits of problem-based and competitive learning. By addressing some of the logistical and practical challenges noted by participants, such events can be further refined to maximize their educational impact, ensuring that future medical professionals are well-prepared with both foundational knowledge and essential clinical skills.

## Appendices

Feedback Form: Dissection Competition - AIIMS Kalyani (2022 and 2023) Department of Anatomy, AIIMS Kalyani

Instructions

Participants are requested to carefully read and respond to the following questions. This feedback pertains to the dissection competition conducted by the Department of Anatomy at AIIMS Kalyani. Fields marked with an asterisk (*) are mandatory.

Consent

1. I am giving my consent for this survey:* ☐ Yes  ☐ No

Dissection Experience

2. Did you find the topic allotted for dissection appropriate? Options:* ☐ Strongly agree  ☐ Agree  ☐ Neutral  ☐ Disagree  ☐ Strongly disagree

3. Time given for dissection was adequate Options:* ☐ Strongly agree  ☐ Agree  ☐ Neutral  ☐ Disagree  ☐ Strongly disagree

4. Cadaver given for dissection was good Options:* ☐ Strongly agree  ☐ Agree  ☐ Neutral  ☐ Disagree  ☐ Strongly disagree

5. Positioning of the cadaver eased the process of dissection Options:* ☐ Strongly agree  ☐ Agree  ☐ Neutral  ☐ Disagree  ☐ Strongly disagree

6. Structures were easily accessible Options:* ☐ Strongly agree  ☐ Agree  ☐ Neutral  ☐ Disagree  ☐ Strongly disagree

7. It was easy to use the dissection instruments Options:* ☐ Strongly agree  ☐ Agree  ☐ Neutral  ☐ Disagree  ☐ Strongly disagree

8. Felt confident before starting the dissection Options:* ☐ Strongly agree  ☐ Agree  ☐ Neutral  ☐ Disagree  ☐ Strongly disagree

9. Identification of the structures during dissection was easy Options:* ☐ Strongly agree  ☐ Agree  ☐ Neutral  ☐ Disagree  ☐ Strongly disagree

Learning Aids & Preparation

10. Using acrylic paint to highlight structures after dissection would help future learning and create good visual memory Options:* ☐ Strongly agree  ☐ Agree  ☐ Neutral  ☐ Disagree  ☐ Strongly disagree

11. Prior reading (theoretical knowledge) before dissecting the area of interest helped during the dissection Options:* ☐ Strongly agree  ☐ Agree  ☐ Neutral  ☐ Disagree  ☐ Strongly disagree

Impact on Learning

12. This dissection competition increased my interest in learning anatomy through dissection Options:* ☐ Strongly agree  ☐ Agree  ☐ Neutral  ☐ Disagree  ☐ Strongly disagree

13. This dissection competition will be helpful for future anatomy examinations Options:* ☐ Strongly agree  ☐ Agree  ☐ Neutral  ☐ Disagree  ☐ Strongly disagree

14. Dissection deepened the understanding of the anatomy of the particular area Options:* ☐ Strongly agree  ☐ Agree  ☐ Neutral  ☐ Disagree  ☐ Strongly disagree

Environment and Future Outlook

15. The smell and effect of formalin (irritation or burning sensation to the eyes) reduced the speed of my dissection Options:* ☐ Strongly agree  ☐ Agree  ☐ Neutral  ☐ Disagree  ☐ Strongly disagree

16. Conducting similar events in future for upcoming batches will be helpful Options:* ☐ Strongly agree  ☐ Agree  ☐ Neutral  ☐ Disagree  ☐ Strongly disagree

17. I shall encourage my juniors to participate in similar events in future Options:* ☐ Strongly agree  ☐ Agree  ☐ Neutral  ☐ Disagree  ☐ Strongly disagree

Open-Ended Feedback

18. How was your overall experience of this competition? Response:* ................................................................................................

19. Suggestions for improvement in future competitions Response:* ................................................................................................

20. Challenges faced during the competition Response:* ................................................................................................
